# Geoarchaeological Evidence of Middle-Age Tsunamis at Stromboli and Consequences for the Tsunami Hazard in the Southern Tyrrhenian Sea

**DOI:** 10.1038/s41598-018-37050-3

**Published:** 2019-01-24

**Authors:** M. Rosi, S. T. Levi, M. Pistolesi, A. Bertagnini, D. Brunelli, V. Cannavò, A. Di Renzoni, F. Ferranti, A. Renzulli, D. Yoon

**Affiliations:** 1 Dipartimento di Scienze della Terra, Università di Pisa, Pisa, Italy; 2 Dipartimento di Scienze Chimiche e Geologiche, Università di Modena e Reggio Emilia, Modena, Italy; 3 Department of Classical and Oriental Studies, Hunter College, City University of New York, New York, USA; 4 Istituto Nazionale di Geofisica e Vulcanologia, Pisa, Italy; 5 Istituto di Studi sul Mediterraneo Antico-CNR, Rome, Italy; 6 Associazione Preistoria Attuale, Bardonecchia, Torino Italy; 7 Dipartimento di Scienze Pure e Applicate, Università di Urbino, Urbino, Italy; 8 American Numismatic Society, New York, USA

## Abstract

Large-scale landslides at volcanic islands are one of the most dangerous geological phenomena, able to generate tsunamis whose effects can propagate far from the source. However, related deposits are scarcely preserved on-land in the geologic records, and are often difficult to be interpreted. Here we show the discovery of three unprecedented well-preserved tsunami deposits related to repeated flank collapses of the volcanic island of Stromboli (Southern Italy) occurred during the Late Middle Ages. Based on carbon datings, on stratigraphic, volcanological and archaeological evidence, we link the oldest, highest-magnitude investigated tsunami to the following rapid abandonment of the island which was inhabited at that time, contrary than previously thought. The destructive power of this event is also possibly related to a huge marine storm that devastated the ports of Naples in 1343 (200 km north of Stromboli) described by the famous writer Petrarch. The portrayed devastation can be potentially attributed to the arrival of multiple tsunami waves generated by a major landslide in Stromboli island, confirming the hypothetical hazard of these phenomena at a regional scale.

## Introduction

**Figure 1:**
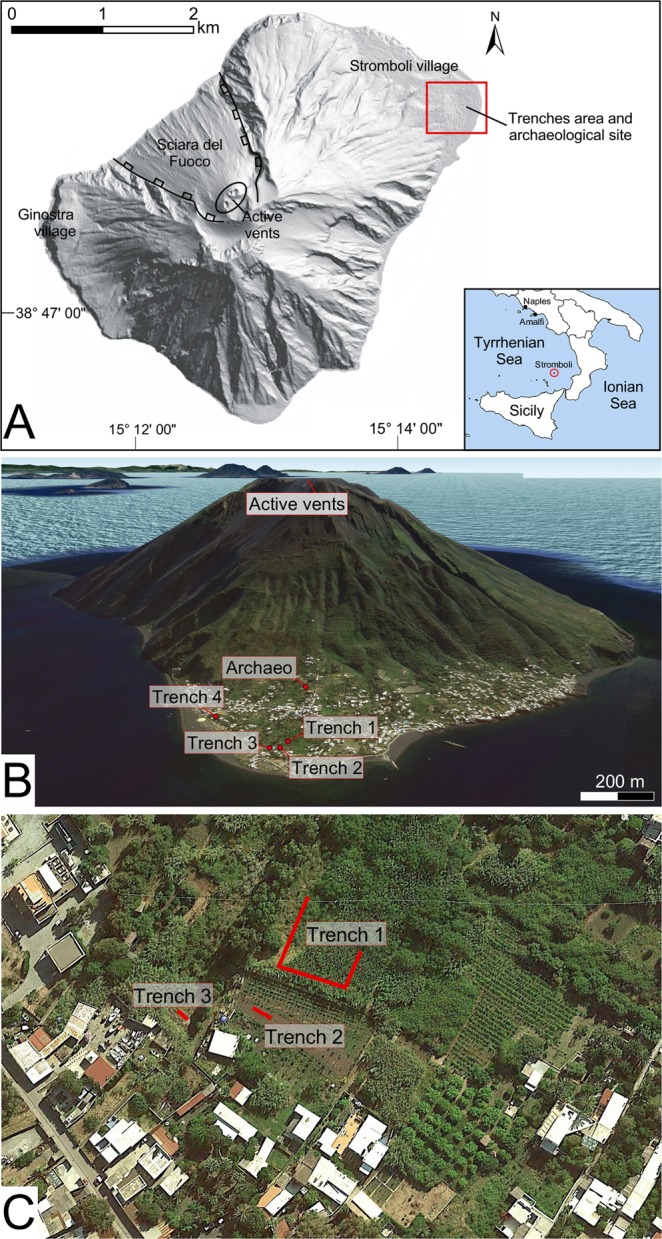
(**A**) General map of Stromboli showing the studied area in the red box. Inset shows the localization of Stromboli in the Tyrrhenian Sea. (**B**) Aerial view, from north to south, of Stromboli (Google Earth image) with location of trenches and of the archaeological site of San Vincenzo. In the background, other islands of the Aeolian Archipelago and Sicily are shown. Inset show the localization of Stromboli in the Tyrrhenian Sea. (**C**) Detail of the area close to the coastline, with location of the trenches (Map data: SIO, NOAA, U.S. Navy, NGA, GEBCO, TerraMetrics, ^©^2018 Google).

Human communities can be subjected to catastrophic migrations triggered by anthropogenic and/or climatic factors (wars, economic crises, climate change) and for safety reasons due to natural hazardous phenomena. The island of Stromboli, located in the Aeolian archipelago (Southern Italy; Fig. 1A), has exerted, since ancient times, a strong appeal due to its mild climate, fertile soils, and strategic position controlling the nautical routes of the Tyrrhenian Sea. However, the prosperity of the settlements on the island since the Late Neolithic had to periodically face the threat of the volcano whose craters are located less than two kilometers from the coastline. During the last centuries, numerous eruptive crises frightened the inhabitants and severely hit the settled areas^1^. It is therefore possible that the island was repeatedly abandoned in the past due to the insecurity and physical impacts induced by the activity of the volcano.

A coordinated archaeological-volcanological research project was launched in 2016 to investigate the events that took place on the island in the Late Middle Ages. We demonstrate that a significant human settlement was present on the island in the early 1300 s and that this community suddenly left the island, after the first half of the fourteenth century, due to catastrophic events related to volcanic activity and flank failures of the volcanic cone. Based on carbon datings and archaeological evidence, we also speculate that historical documents describing huge marine storms and devastation in the far-field (Naples and Amalfi, 200 km north of Stromboli island), can be potentially attributed to the arrival of tsunami waves generated at Stromboli. While for the two youngest tsunami events the attribution is not conclusive, the poorly understood, sea storm witnessed in the port of Naples by the writer Petrarch (Francesco Petrarca) on November 25, 1343^2^, was likely attributable to the oldest discovered tsunami whose origin can be placed at Stromboli and which induced the rapid abandonment of the island.

## Results

### Volcanological results

An in-depth analysis of deposits accumulated in the NE sector of Stromboli island was undertaken with the goal at investigating the possible presence of traces of significant volcanic and tsunami events occurred in recent times. For this purpose, three trenches (Trench 1 to 3; Fig. 1B,C), 1.5 m wide, about 2 m deep and with a cumulative length of about 80 m, were dug. The three trenches align roughly with the active craters and the archaeological site of San Vincenzo. They were located at distances of 170 m to 250 m from the present shoreline in an area of flat, gently inclined topography, and at a height above sea level (a.s.l.) between 2 and 6 meters. A fourth trench (Trench 4), previously excavated during the 2000s about 200 m south, was reopened for comparison and sampling (Fig. 1B,C).

**Figure 2:**
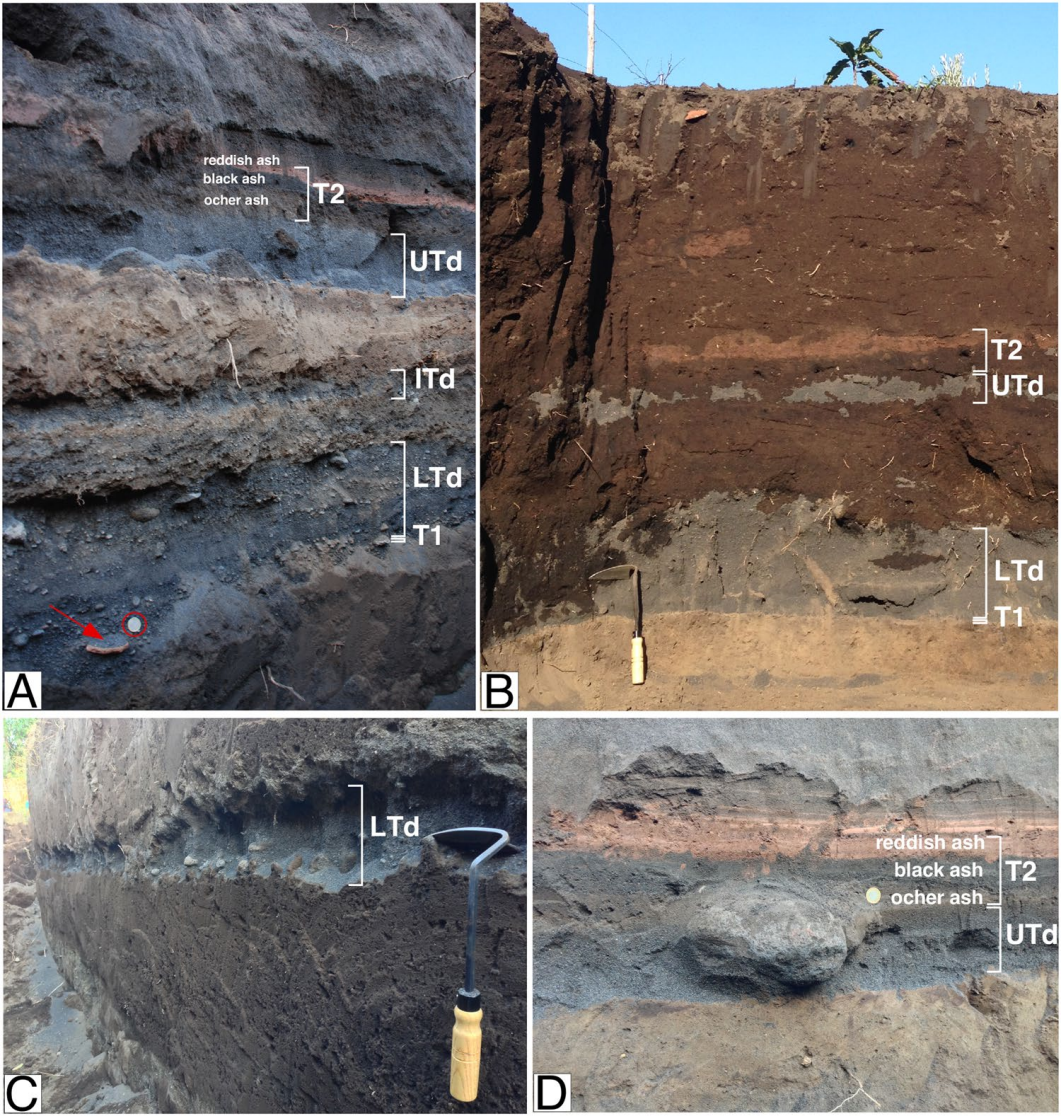
Pictures showing the tephra and tsunami sequence in (**A**) Trench 3, (**B**) Trench 2, and (**C**) Trench 1. (**D**) A detail of Upper Tsunami deposit (UTd) and T2 tephra from Trench 2. Tool for scale is 30 cm. One Euro coin in (**A**) for scale is 23.25 mm. The red arrow in (**A**) indicate a ceramic tile. T2 consists of two, red and black ash to lapilli tephra beds and of an ocher-colored bed, related to deposition of tephra from explosive activity at summit craters together with dust fallout from landslide activity.

Both the position of the trenches and the explored depth interval were effective in bringing to light the main volcanic and marine events of the past 1000 years. The three trenches crossed a succession of well-structured, unconsolidated sediments with some individual beds showing excellent lateral continuity (Fig. 2). The most prominent feature of the succession was the presence of three beds that we interpret as tsunami deposits based on their striking sedimentological characteristics^3,4^. The three beds are composed of loose, crystal-rich, well-sorted, black sand, embedding rounded clasts of lava (beach pebbles/cobbles). These beds were found to overlie, via sharp, locally erosive contacts, massive, fine-grained deposits of continental origin consisting of weakly altered, fine-grained, airborne ash. The bed-forming sandy and pebble materials show spectacular similarity in composition and color with the present-day beach sediments (backdrop near the shore, beach and coastal dune).

The lowermost bed (LTd) is the thickest, coarser-grained and most widespread. The thickness of the bed is around 40 cm in the trench closest to the present coastline and gradually decreases to 5 cm moving away from the coast to the higher sites, over a distance of 70 m. A similar gradual decrease from 17 cm to 2 cm is observed in the equivalent diameter of individual lava clasts. The nature of bed-forming materials, thickness and grain-size features, and related inland variations of the grain-size parameters are all consistent in indicating an origin from transport of the materials away from the shore by a high-energy tsunami wave. A further relevant feature of LTd deposit in the trench nearer to the present shoreline, is the presence of two finer-grained, lower, normally-graded, sub-beds and a coarser, reversely-graded, locally clast-supported upper sub-bed (Fig. S1). The sequence suggests the tsunami event consisted of multiple waves, occurred in succession with the last one having greater energy.

The intermediate sandy bed (ITd) only occurs in the trench nearest to the coast, where it has a thickness of 4 cm. It is composed of loose black sand and sporadic rounded cm-sized pebbles (Fig. 2). The upper sandy bed (UTd) has again similar lithologic features and an analogous decrease of thickness and pebble diameter moving from the coast inland. It can be traced up to 220 m from the present shoreline, showing intermediate grain-size and thickness characteristics between LTd and ITd.

The three deposits are not compatible with storm events because the east coast of the island is exposed to waves of small height due to the limited, 50 km-wide, marine fetch which separates Stromboli from the eastern and southern coasts of Italy (Fig. 1). On the other hand, the sharp contact between the continental fine-grained ash and the sandy deposits also excludes that beds could be the result of erratic shoreline migrations (sea transgression-regression cycles).

It is worth to note that the three tsunamis were by far more energetic than any event that occurred in the last two centuries at Stromboli^5,6^ as we know from deposit studies and descriptions of the historical activity.

In addition to the tsunami beds, we identified two primary, lapilli-bearing, ash fallout layers (tephra) immediately below the LTd and at the top of UTd deposits. The two tephra layers were respectively labeled as T1 and T2 (Figs 2 and S2). T1 consists of a 1 cm-thick, fine ash fallout deposit, which embeds at the base cm-sized, banded, light and dark-colored pumice lapilli. The ash fallout is locally separated from LTd by 2–3 cm thick, fine-grained, reworked sedimentary material embedding small charcoal fragments (Figs 2 and S1). The reworked sediments suggest that the explosive eruption that caused the deposition of T1 occurred sometime before (months to years) the tsunami event.

Tephra T2 consists of a bed-set including, from base to top: (i) a 4 cm-thick, ocher-colored bed of fine to medium ash; (ii) a 2–3 cm thick bed composed of black, shining, coarse ash to fine-grained glassy lapilli, with sparse clasts of banded light/dark-colored pumice at the base; (iii) an upper 5–6 cm thick, hardened, reddish-brown bed composed of altered and oxidized fine ash (Figs 2 and S1).

The T2 tephra bed-set directly overlies the unaltered and not reworked sand of UTd indicating that a short time lapse separated the tsunami from T2 (Fig. S1). Microscopic examination of the lowermost ocher-colored ash material revealed that the ash grains consist of coarse-grained fresh juvenile component mixed with fine-grained lithic altered material. Such features are consistent with the deposition of tephra from explosive activity at summit craters together with dust fallout from landslide activity. The lack of intra-bed reworking indicates that UTd, and the entire T2 tephra bed-set were all emplaced in rapid succession within a short time interval (days to months).

Tephra T1 and T2 exhibit features typical of the largest explosive events of the present activity of the volcano (paroxysmal explosions)^1,7–9^. From the record of the past two centuries it is well known that these events are capable of producing cm-thick accumulation of tephra up to the coast line, local shower of ballistic blocks, arrival of powerful shock waves, and ignition of wildfires in the vegetation^1,10,11^.

The ages of the volcanic and tsunami deposits crossed by the trenches were constrained by means of three ^14^C dates on charcoal fragments found directly below the tsunami and tephra deposits. A charcoal fragment below LTd (sample St16-82) gave a calibrated 2σ (95.4%) age of 1224–1298 AD (Table S1). Dating of a charcoal fragment at the base of UTd (St16–34) gave a calibrated age of 1426–1515 AD. Dating of T2 bed-set from a charcoal fragment embedded in the ocher-colored ash bed (St16-71) yielded a calibrated age of 1477–1642 AD. Although ^14^C ages have ∼one century of uncertainty among tephra T2 and UTd, field observations suggest the age of the two events to be almost identical (separated by days to months).

### Archaeological results

The Aeolian Archipelago has been inhabited since the 6^th^ millennium BCE, and human occupation of peripheral Stromboli started during the 4^th^ millennium BCE. The most complete archaeological dataset comes from the site of San Vincenzo, located near the northern end on a large plateau (about 6 ha; altitude 40–100 m a.s.l.). This location provides a remarkable visual control over the southern Tyrrhenian Sea and its entrance from the strait of Messina^12^.

The site has been investigated since 2009 with a multidisciplinary approach^13–16^. The excavation extends over 700 m^2^ over three main areas (North, East, and West) on an irregular, sloping surface (Fig. S3). Archaeological evidence attests human presence during the Late Neolithic, Chalcolithic, Bronze Age, Hellenistic, Late Roman, and Medieval periods^17,18^. Medieval occupation on Stromboli was previously unknown, though it is known for Lipari, revealing a completely new settlement and occupation scenario.

The medieval history of the Aeolian Islands was closely tied to that of Sicily. After the Byzantine Empire and the Arab Emirate of Sicily (6^th^–11^th^ cent.) the Normans conquered and ruled Sicily, the Aeolian Islands, and southern Italy in the late 11^th^ cent. Due to a succession dispute in the 13^th^ cent., Sicily was politically divided from the mainland for two centuries: the dynasty of Aragon ruled Sicily and the Aeolian Islands, while the family of Anjou ruled mainland southern Italy. During this long period of conflict, Sicily experienced economic difficulties, made worse by the Black Death of the mid-14^th^ cent. In the 15^th^ cent., Sicily and southern Italy were once again united under the Kings of Aragon, and later Spain.

**Figure 3:**
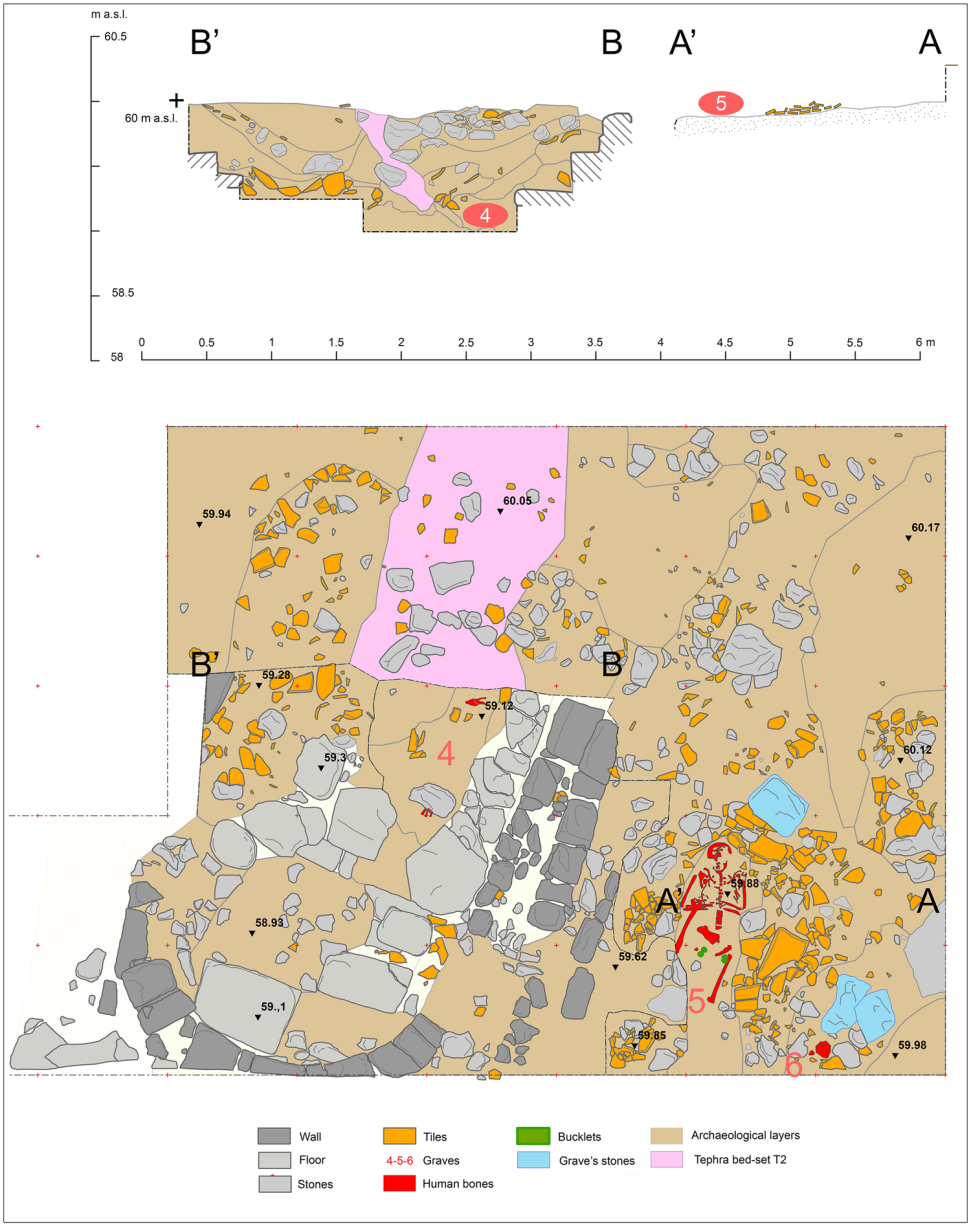
Excavation wall (vertical face; above) and plan (below) of the medieval building and graves (n. 4–6) in the area north of the archaeological excavation. Graves found within the religious building consist of shallow pits dug through the layer of collapsed tiles, suggesting that burials occurred immediately after its collapse.

At San Vincenzo, medieval buildings and graves have been discovered in the North area (Fig. 3), and a medieval channel filled with architectonic rubble is attested in both East and West areas (Fig. S3). Both the medieval buildings and the channel are lined by a tephra sequence consisting of: (i) an ocher-colored ash layer; (ii) dark-gray ash layer with sparse pumice lapilli; and (iii) 2–5 cm-thick, reddish-brown ash layer (Figs 3 and S4–S6). Thickness, grain-size and components of individual beds forming the tephra sequence are identical to those shown by tephra T2 observed in the trenches along the coast, allowing an unequivocal correlation among them.

**Figure 4:**
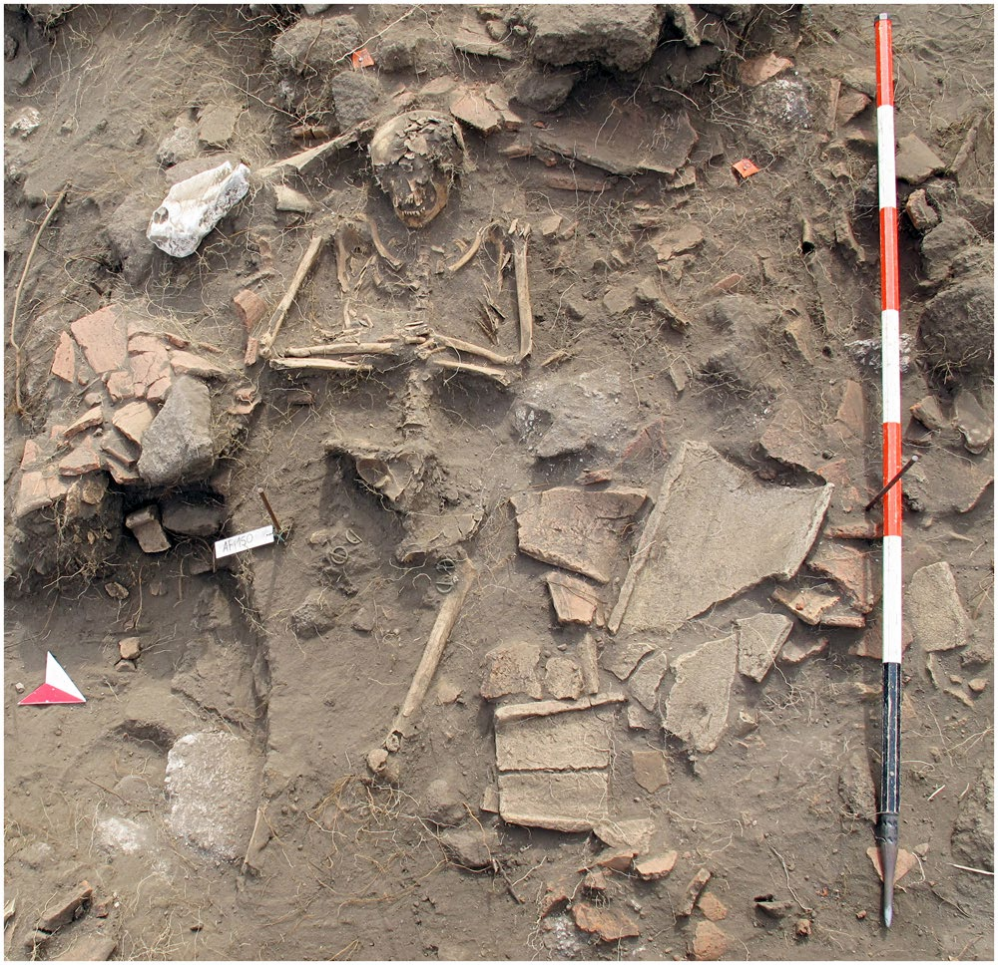
Photo of the grave 5 in the area North of the archaeological excavation. Each bar on the scale is 20 cm.

The main medieval building is a small church with a tile roof and a semicircular stone apse, large paving stones, and an adjoining room on its northern side. Three graves were discovered in the apse and the northern room (Fig. 4). The apse area is characterized by a complex concave stratigraphy, with holes and pits caused by post-depositional processes that partly disturbed the original sequence of layers, in turn sealed by tephra T2 (Fig. 3). Noteworthy, for the purposes of precise dating, is the discovery of numerous coins.

The chronological reconstruction is based on chrono-typological and radiometric evidence. Thermoluminescence dating on tiles (Table S2) gives a range for their production. A tile from the apse is older (1080–1180 AD) while one from the northern room is more recent (1270–1350 AD). Petrographic analyses of tiles and other ceramics show imports from Sicily or Calabria as well as local products^19^. Some tiles are decorated with finger-impressed wavy lines (Fig. S7), a style with parallels in southern Italy dated to the 11^th^–13^th^ cent^20,21^.

Remains of three individuals have been discovered, two of which have been dated by ^14^C. Two were buried in a room immediately north of the apse (graves 5 and 6), oriented west-east with the head toward west and a stone nearby indicating the position (Fig. 3). The grave pits were dug through the layer of collapsed tiles; this stratigraphic position suggests the burials occurred immediately after collapse of the building. The ^14^C calibrated 2σ range for individual 5 is 1295–1404 AD (sample 1769 in Table S1). Grave 4 was located in the apse in connection with a gap of the stone floor paving, the body being only partially preserved. The ^14^C calibrated 2σ age of the bones is 1311–1434 AD (1757 in Table S1) and that of charcoal found in the sediment filling the hole is 1285–1400 AD (1759 in Table S1).

Four annular buckles were found in close association with the skeleton of grave 5 (Fig. S8). They appear to have been fasteners for some dress accessory such as a complex belt, worn by the person buried in the grave. All four are very similar in shape, size, and material and can be dated to the late medieval period from the late 13^th^ to 14^th^ cent. (Figs S8, S9)^22^.

Nineteen identifiable medieval coins^23^ provide the most specific information about the chronology of medieval occupation: they were produced between 1266 and 1402 at maximum (Fig. S10). The earliest are two denari of Charles I of Anjou, who was king of Sicily from 1266 to 1285. The latest coin is a denaro from the joint reign of Maria and Martin I of Sicily (1395–1402). The two coins of Charles I were struck before he lost the control of Sicily in 1282, so the minimum possible chronological range for these coins is 1282 to 1395. Most of the coins were produced before 1343; however, four coins were certainly produced after 1343. The abundant Sicilian coinage of Frederick II (1198–1250) has not been found at San Vincenzo so far, and the abundant Sicilian coinage of Alfonso (1416–1458) has also not been found^24^.

The tephra sequences in the church apse and in the channel give us the opportunity to define the range between the collapse of the building/formation of the channel and the deposition of the tephra layer, with several ^14^C dates on charcoal from the stratigraphy. In agreement with other evidence, the base of the channel is dated 1325–1445 AD (265 in Table S1) and a layer between tiles and tephra at the end of the 14^th^-beginning of 15^th^ cent. (1390–1440 AD; 1788 in Table S1). The series of dates obtained immediately below the tephra are all around the 15^th^ cent (Table S1).

## Discussion

**Figure 5:**
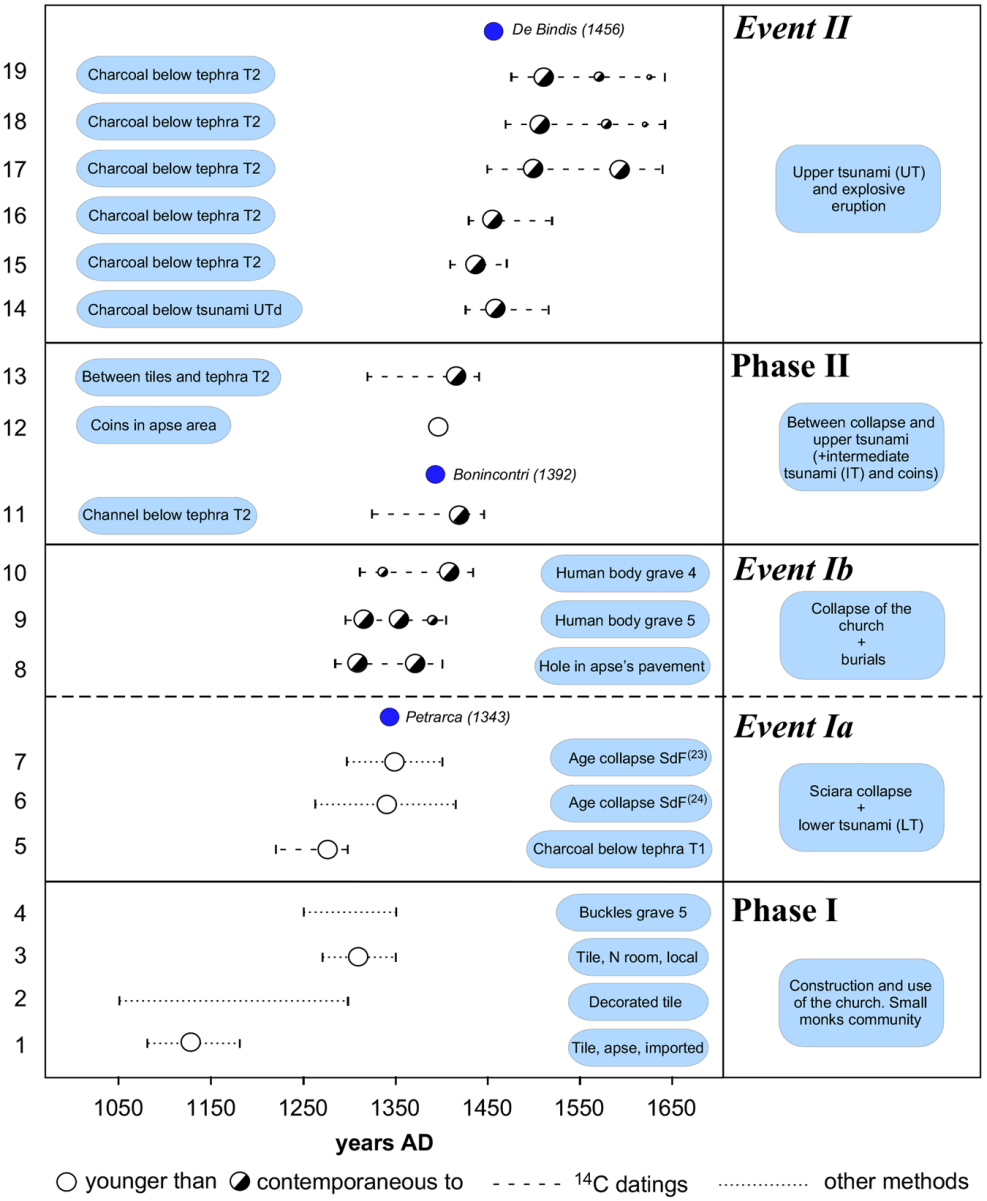
Synthetic chrono-stratigraphical diagram with evidence collected in the trenches and in the archaeological site. Age control is from eleven ^14^C ages, two thermoluminescence ages, and two archaeo-paleomagnetic ages^25,26^. In addition, dates from recovered material includes two coin ages, one from the usage of a belt buckle found in the grave 5, and one by the decorative style of the tiles. The mean values of ^14^C ages having more than one intercept with the calibration curve are graphically represented with circles of different diameters for different probabilities. The year of suspected occurrence of tsunamis through letters and past chronicles is also reported with blue dots: 1343 event by Petrarch^2,31^, 1392 event described by L. Bonincontri^2^, 1456 event by B. De Bindis^32^.

All the most relevant chronological information, ^14^C and archaeological ages along with previous paleo-magnetic dates^25,26^, are plotted in Fig. 5. The five vertical blocks in which the chronological information is organized (Phase I, Events Ia and Ib, Phase II and Event II) reflect their stratigraphic position based on the correlation with T2 tephra. The data provide a tight temporal constraint for human and natural events on Stromboli in the time interval between 1200 and 1500 AD. With respect to Event II, the ages consist of ^14^C ages obtained on charcoal fragments collected immediately below the UTd in the trenches and directly below the T2 tephra in the archaeological site. The correlation of T2 tephra bed-set between the two areas is unequivocal, so all of the ages constrain the same composite event.

Archaeological evidence and relative chronological constraints enable us to establish that in the Late Middle Ages, a significant human community was present on the island during the Crusades period likely due to the strategic position of Stromboli on the naval routes heading from Europe to the strait of Messina (Phase I; Fig. 5). This can be deduced from the presence of a religious building with materials (tiles and pottery) imported from Calabria/Sicily after the 12^th^ cent. The excavation of the building also established that the structure suddenly suffered a traumatic, never restored, collapse of the roof. The fact that the graves found within the religious building consist of shallow pits dug through the layer of collapsed tiles, suggests that burial of bodies occurred immediately after the collapse itself and was either made with haste or with a symbolic value (Event Ib). After the collapse of the building, the presence of humans on the island is diminished (Phase II). While the occurrence of medieval coins slightly younger that the most probable collapse age may indicate that a few people remained or occasionally visited the island until at least the late 14^th^ century, the lack of reutilization of the building material (a common practice at that time) suggests a sudden and prolonged abandonment of the site. In large parts of the excavation, the layer of collapsed tiles lies almost intact, undisturbed with the exception of the graves, impregnated with aeolic volcanic ash and ubiquitously sealed by the T2 tephra of the 15^th^ cent. (Event II). During this period (Phase II and Event II, i.e. between the building collapse and the emplacement of T2 tephra), the two other tsunami deposits can be placed. Based on ^14^C dates, they can be conveniently placed in a time span between 1350 and 1500.

One of our most relevant discoveries is that the paroxysmal explosion (T1), the major tsunami event (LT), the collapse of the religious building, and the death of individuals are virtually contemporaneous. Our work thus raises the question if and how the natural events were somehow responsible for the church collapse. The minimum age of the LT tsunami event is the end of 1200 s. During the period 1200–1400 no regional earthquake with M_w_ > 6 is reported in the coastal region of Southern Italy^27^. Although historical catalogues are characterized by a large degree of incompleteness for the Middle Ages, a seismic origin of the tsunami is unlikely. Indeed, the chronological vicinity of the charcoal age below the LT and that of lavas truncated by the most recent (1350 ± 60), large-scale collapse of the Sciara del Fuoco^25,26,28,29^ is suggestive of a cause-effect relationships between the collapse and the tsunami event. Such an interpretation is also in agreement with the generation of several waves recorded in the deposits possibly due to multiple rock detachments along the volcanic flank. Regarding the cause of the collapse of the church’s roof, this could have been produced by either ash accumulation or ballistic fallout produced by the violent T1 paroxysmal explosion or by seismic shaking. During excavation, we did not find conclusive evidence indicating ash accumulation or ballistic fallout. Shaking during the landslide with enough intensity to cause the collapse of the building cannot instead be discarded. The possible rock volume involved in the flank collapse may in fact have exceeded ~1 km^3^, as suggested for all the <5 ka tsunamigenic lateral collapses occurred at Stromboli based on the geometry of the observed scarps^28^. A recent review of the seismic effect of large-scale landslides^30^ has in fact shown that landslides with volume on the order of 1 km^3^ have the potential of producing local seismic shaking capable of inducing collapse of weak roofing stocks. A local, shallow, low to moderate seismic event possibly responsible for both church’s collapse and the Sciara del Fuoco landslide, cannot be excluded.

Based on geological, archaeological and carbon datings, the three tsunami deposits can be placed in a time interval between fourteenth and sixteenth centuries. In the same time interval, three suspected tsunami events recorded along the coasts of Campania and described in historical chronicles^2,31,32^ are reported in 1343, 1392, and 1456 (Fig. 5 and Supplementary Material). The three events have ages that remarkably agree with the time window of the three tsunami events discovered at Stromboli in this work. Recent, far-field observations of the 2002 tsunami generated from a Stromboli flank collapse demonstrate its possibility of reaching the Southern Campania region with in-land propagation of 60–70 m^33^. The largest and oldest event of Stromboli is in agreement with the description of a special witness, the famous writer Francesco Petrarca (1304–1374), who in that moment was in Naples, host in the convent of the friars of S. Lorenzo, as ambassador of Pope Clemente VI sent to the Regency Council of the kingdom. Petrarch, in a letter written on November 26^th^, 1343, to Cardinal Giovanni Colonna^2,31^, describes an impressive, unprecedented sea-storm, which occurred in the night between November 24^th^ and 25^th^, causing hundreds of deaths in the harbor of Naples and destroying several boats both inside and outside the harbor. This same event had a regional impact on all the coast of Campania; in particular it largely wiped away the port structures of Naples and Amalfi^2,31,34^ that are directly exposed to waves generated in the Aeolian region. Although the letter by Petrarch and the devastation of Amalfi have been interpreted in various ways^2^ (a tsunami caused by submarine eruption or by an offshore earthquake or by a landslide on Mt. Epomeo at Ischia’s island), with some of them being unlikely^34^ (extraordinary storm, mudflows), these include the possibility that a tsunami struck the Campania costs on 24^th^ November 1343. Based on our findings, a possible correlation with the LT event may be formulated.

The youngest most important tsunami event that according to^32^ affected the Southern Tyrrhenian Sea is that of 1456. Although the information on the tsunami is not precise, this source clearly report that it struck the port of Naples causing severe damage. Our reconstruction of the UT tsunami recorded on the island of Stromboli indicates that timing and scale of the event are well compatible with historic descriptions^32^. The 1456 tsunami remarkably occurred the same day (5 December) of the largest historic earthquake of peninsular Italy (M_w_~7.0), which caused massive destruction in Campania and Southern Italy^35^. However, we propose for the triggering of the tsunami a large-scale collapse of the Sciara del Fuoco rather than this earthquake. This is based on several lines of evidence: firstly, the presence of the ash deposited right after the UT event which we interpret as due to a prolonged landslide; secondly, the occurrence in close contemporaneity of the T2 paroxysm possibly triggered by pressure release in the magmatic system; thirdly, because the earthquake occurred on land, and the trigger of a tsunami event is unlikely. Although the two events (landslide-triggered tsunami at Stromboli and the Southern Apennines earthquake) may have been unrelated, we could speculate that the large regional earthquake was the possible trigger of the Sciara del Fuoco destabilization and collapse. The triggering capacity of high-energy, long-lasting seismic shaking to produce large-scale landslides is shown by a review of the problem at global scale^36^. If we consider a regional earthquake of magnitude M_w_ = 7.0^35^ and a distance of ~230 km equivalent to that from the epicenter and Stromboli, we are within the upper bounds of maximum epicentral distances of disrupted landslides related to earthquake magnitude as calculated by^36^. The shaking produced by the earthquake cannot be excluded as a possible cause for the Sciara collapse. As it regards the tsunami event of 1392 (IT), although our information is very limited^2^, the absence of a contemporaneous, high magnitude regional earthquake possibly suggests a similar local origin from a Stromboli landslide. The time bracket obtained for this event is consistent with this possible tsunami, although the assignment of a precise age to the discovered IT deposits is not conclusive.

## Conclusions

The major achievements of our work are twofold. First, we reveal that the abandonments of the island during the Middle Age represents a case history of how repeated adverse natural phenomena were able to overcome the resilience of a small human community. The lack of resilience probably derived from the combined factors of living in a small island with the inability to overcome the superposed effects of ground-shaking and coastal tsunamis. Such inability resulted in the complete abandonment and relocation to areas deemed safer. Stromboli is an iconic place where, as expressed by the poet Joseph Brodsky, “geography provokes history”.

Second, we bring to light that the present community of Stromboli, together with the communities now settling the southern coasts of the Tyrrhenian Sea, are actually exposed to a much higher tsunami hazard than previously thought. At local scale, hazards posed by the collapse of the entire Sciara del Fuoco would include the impact of tens of meters-high waves, as well as exposure to destructive seismic shaking possibly resulting from the landslide itself. At larger scale, and if our combination of geological, archaeological and carbon datings with the possible tsunami events described in the chronicles is correct, the height of expected tsunami waves is able to reach and devastate the present port structures and settled coasts of Campania, Calabria, and Sicily. Although modeling results based on a scenario of 1-km^3^ collapse of the Sciara del Fuoco presented by^37^ only discussed the impact along the coasts of Sicily and Calabria, similar effects may be qualitatively expected along the Campanian coasts located just north of Calabria. The discovery that three catastrophic collapses of the Sciara occurred recently and in a short time interval points out that the hazard associated with these events is greater than previously known. Moreover, the morphological and eruptive framework during the Middle Ages is similar to the present, suggesting that a similar scenario cannot be ruled out today. This work also demonstrates that frequency of the flank collapse of Stromboli, maximum volume of the collapses, and combined occurrence of flank collapse and large-scale eruptions need to be urgently explored for a thorough assessment of hazards at the regional scale.

## Methods

The excavation of the archaeological site was undertaken and documented using the technique of stratigraphic excavation in a 1 × 1 m topographic grid system, 3D rendering, rigorous and systematic classification of the finds and making use of a wide spectrum of specialized scientific techniques. For the study of volcanic and tsunami events, the classical methods of the tephro-stratigraphic analysis were adopted together with those recently employed for the identification and documentation of the on-land, paleo-tsunami deposits. Stratigraphic trenches, excavated by means of mechanical machinery, allowed to identify the presence of previously unknown large-scale tsunami deposits. Accurate altimetry determination of tsunami deposits, were obtained through high-precision topographic survey.

For the chronological reconstruction of human and volcanic events, a combination of various (numismatic, archaeological, typo-chronologic, ^14^C, thermoluminescence, archaeomagnetic) methods was used whereas tephro-stratigraphic correlation techniques were employed to assess isochrones between the archaeological site and the trenches dug near the coastline. Twelve ^14^C dates were made on carbonized vegetable fragments and human finds within the archaeological site and carbonized fragments recovered from in stratigraphic trenches (Table S1). ^14^C radiometric dating was performed using acceleration mass spectrometry (AMS) technique in part at the Beta Analytic laboratories (USA) (https://www.radiocarbon.com/iso-certified.htm) and partly at the laboratories of Milano Bicocca University (Italy). The dating of the tiles was carried out using thermoluminescence measurements^38^ conducted at the laboratories of Milano Bicocca University (Italy) and are the average of 2/3 tiles sampled *in situ* with sediment (Table S2).

## Supplementary information

Supplementary Information
