# Supplementary material for: Geoarchaeological Evidence of Middle-Age Tsunamis at Stromboli and Consequences for the Tsunami Hazard in the Southern Tyrrhenian Sea
Source: Sci Rep. 2019 Jan 24;9:677. doi: 10.1038/s41598-018-37050-3 (PMC6346119; doi:10.1038/s41598-018-37050-3)
Supplement: Supplementary file 1 — Supplementary Information [file 41598_2018_37050_MOESM1_ESM.pdf]

Supplementary Information for

Geoarchaeological Evidence of Middle-Age Tsunamis at Stromboli and Consequences for the Tsunami Hazard in the Southern Tyrrhenian Sea

M. Rosi, S.T. Levi, M. Pistolesi, A. Bertagnini, D. Brunelli, V. Cannavò, A. Di Renzoni, F. Ferranti, A. Renzulli, D. Yoon

Correspondence to: mauro.rosi@unipi.it, sl1889@hunter.cuny.edu

Supplementary Text

Translation of a Letter by F. Petrarca

Francesco Petrarca (1304-1374), host in Naples in the convent of the friars of S. Lorenzo, as ambassador of Pope Clemente VI sent to the Regency Council of the kingdom. Petrarch, in a letter written on November 26, 1343, to Cardinal Giovanni Colonna.

"[...] As for me, if ever there is a sufficient block of time, the Neapolitan storm [in Latin: 'tempestas'] can provide me with abundant material for a poem, althought it was not only Neapolitan but spread throughout the upper and lower seas [in Latin: 'totius Superi atque Inferi Maris', i.e. Adriatic and Tyrrhenian seas] and according to hearsay was almost universal. [...]

[...] It would take too long to try to describe in words every horror that surrounded that infernal night, and although my words may fall short of the truth, they will tranced any plausibility that can be placed in truth itself. What a downpour! what winds! what lightning! what deep thunder! what frightening tremors! what roaring of the sea! what shrieking of the populace! In this state which made the evening as if by magic appear twice as long, when we finally began to glimpse the dawn, the daylight appeared imminent rather through conjecture than in actual fact. The cloaked priests repeated their sacrifices at the altar and we, not daring to look at the heavens, threw ourselves prostrate on the moist and naked floors around them. When no doubt remained that it was indeed daylight (though it continued to resemble the glow of night) and the shouting of the populace suddenly became silent in the upper part of the city, although it seemed to be increasing more and more from the direction of the shore, and we could not learn what was happening by inquiring, our despair, as often happens, became boldness, and we mounted our horses and descended to the port determined to see for ourselves and to perish if necessary.

Good God! When was anything like this ever heard of? The oldest sailors asserted that what had happened was indeed without parallel. The port was filled with frightening and dismal wreckage. The unfortunate victims, who had been scattered by the water and had been trying to grasp the nearby land with their hands, were dashed against the reefs and were broken like so many tender eggs. The entire shore line was covered with torn and still living bodies: someone's brains floated by here, someone else's bowels floated there. In the midst of such sights the

yelling of men and wailing of women were so loud that they overcame the sounds of the seas and the heavens. To all this was added the destruction of buildings, many of whose foundations were overturned by the violent waves against which that day respected no bounds and respected no work of man or nature. They overflowed their natural limits, the familiar shoreline, as well as that huge breakwater which had been constructed by zealous men and which, with its outstretched arms, as Maro says, constitutes the port, and all that portion of the region which borders the sea. And where there had been a path for strolling there was now something dangerous even for sailing. [...]

[...] On land we could scarcely find an escape and neither on the deep nor in port could a ship be found to equal those waves. Three long ships from Marseilles, called galleys, which had returned from Cyprus after crossing wide expanses of the sea and were anchored there ready to depart in the morning, we saw overcome by the waves with a universal outcry and without anyone being able to offer assistance and without a single sailor or passenger saved. In the same manner other even larger ships of all kinds which had taken refuge in the port as if in a fortress were destroyed. Only one of so many survived. [...]"

Translation of a Letter by L. Bonincontri

A text by Lorenzo Bonincontri, an astrologer born in San Miniato in Tuscany in 1410.

"[...] In the year of Salvation 1392, the sixth day before the calends of February [January 27], many prodigies occurred in the Kingdom [of Naples]. In fact, in the Neapolitan coast in an extraordinary way, having dried for more than forty steps the waters (50 m), appeared the seabed, and the sea retreated in its depths [...]"

Translation of a Letter by B. de Bindis

Letter written on December 7, 1456 by the Siena ambassador in Naples Bindo de Bindis to the lordship of Siena.

"[...] On day 4 of this (December) at the eleventh hour (3 a.m. of December 5) an earthquake occurred, which lasted a tenth of an hour and perhaps more; and it was so great that all this region is ruined. During the night, there was a great stirring of the sea, so that it seemed that all the galleys and ships that were in the port were fought by a thousand devils, great were the ruin and the flapping of the ships among them, that those who were on board believed to die. A small boat was broken, but by the grace of God no one died and only the cargo was lost [...]"

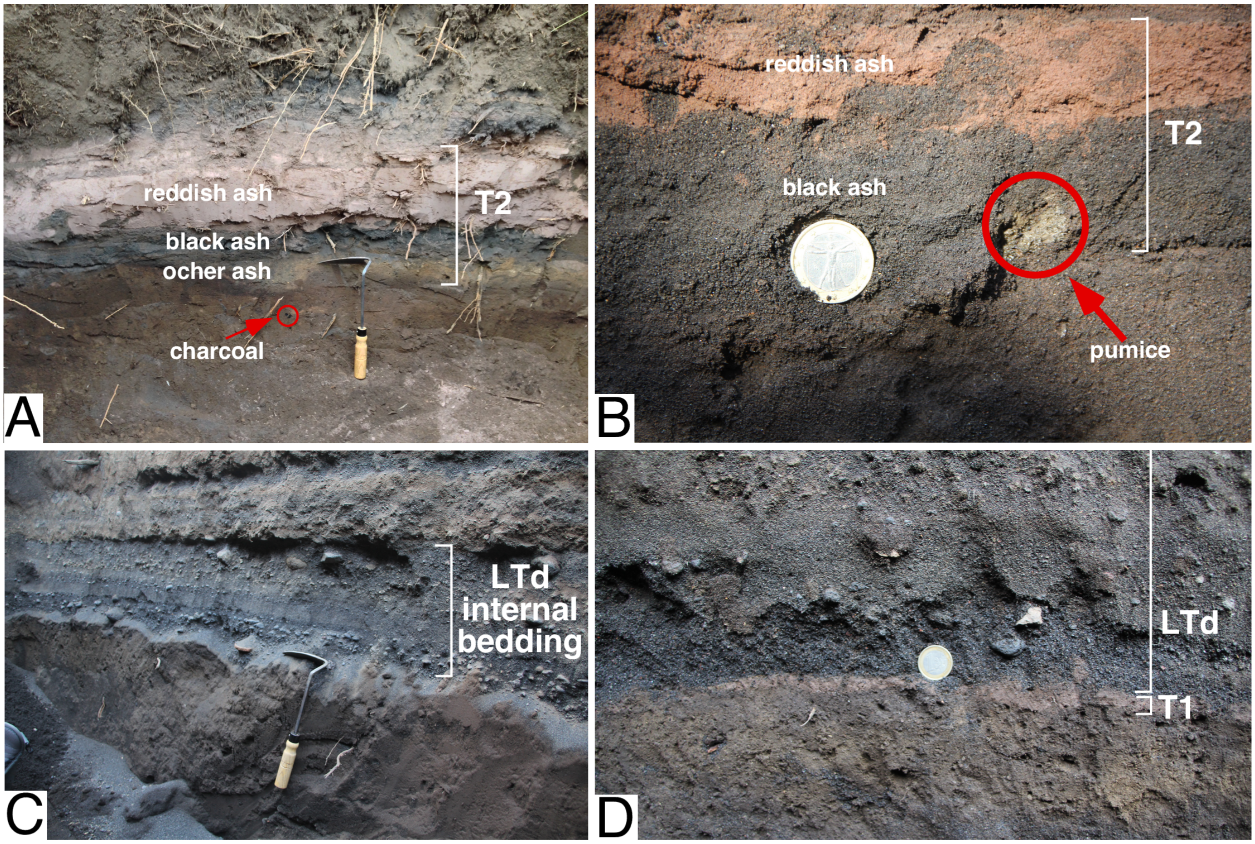

Figure S1.

(A) The T2 tephra sequence of Trench 4. Charcoal in the red circle is indicated by the red arrow. (B) T2 tephra (ocher, black and red ash) in Trench 3. Pumice at the base of the black ash indicated in the red circle. (C) Internal bedding of LTd in Trench 3. (D) T1 tephra below LTd in Trench 3. Tool for scale is 30 cm. Coin for scale is 23.25 mm.

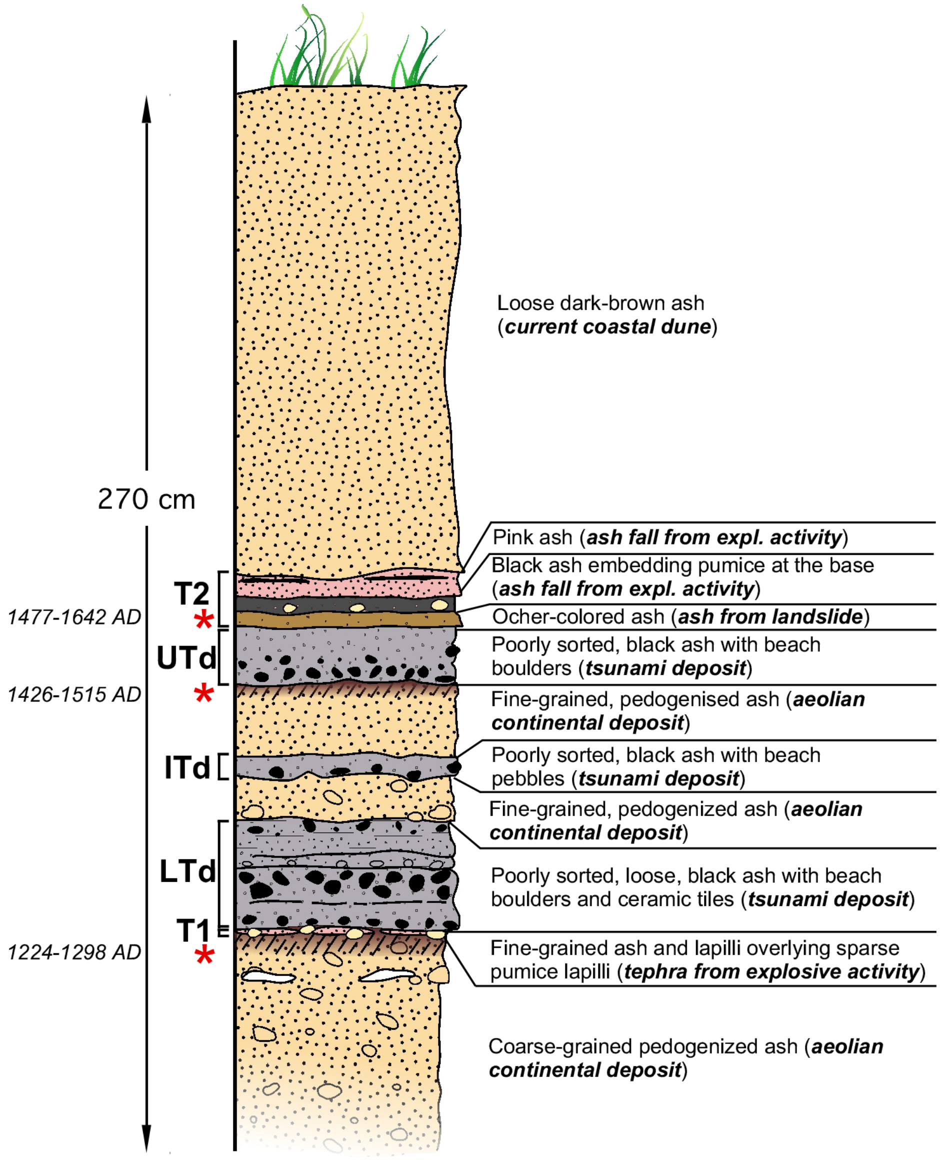

Figure S2.

Ideal composite reconstruction of the stratigraphy of tsunami and tephra deposits of the trenches. Red asterisks refer to dated charcoals.

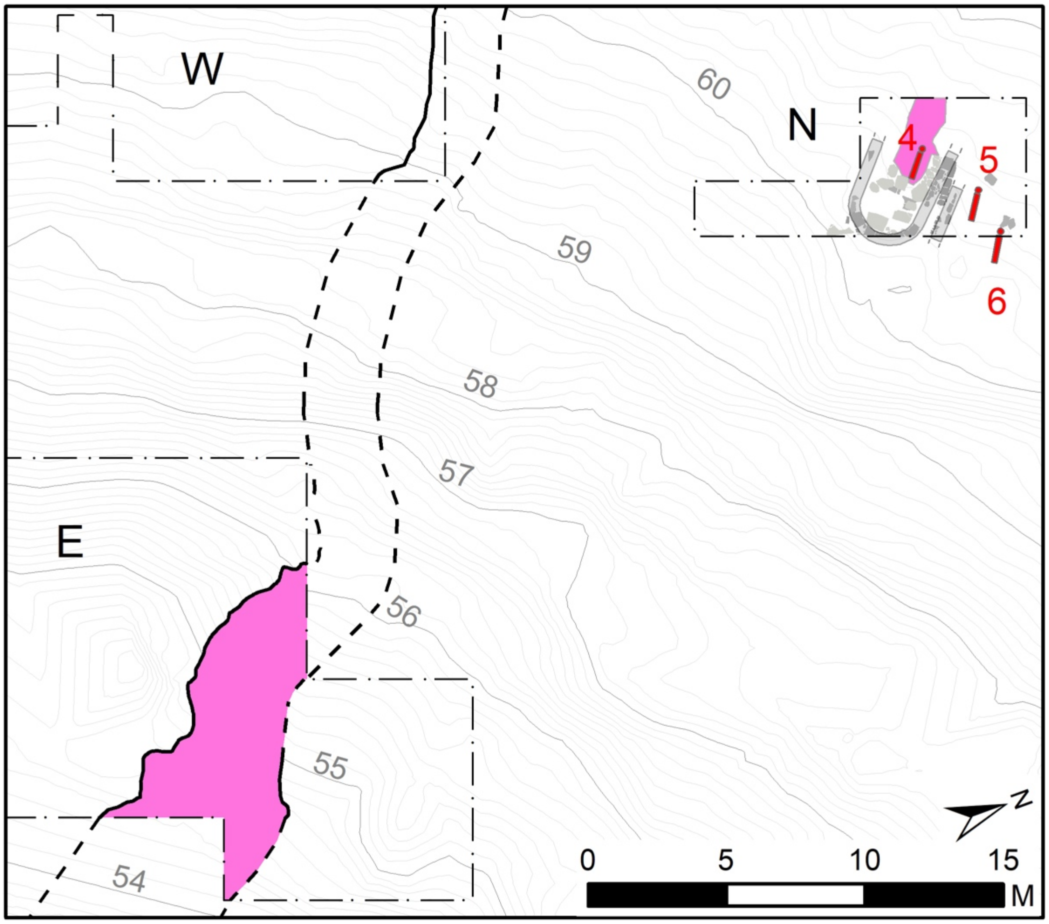

Figure S3.

General map of the archaeological excavation in the North (N), East (E), and West (W) areas with the WNW-ESE channel (center-left), the medieval building (gray), the graves (red, n. 4, 5, 6) and the well-preserved tephra T2 layers (pink).

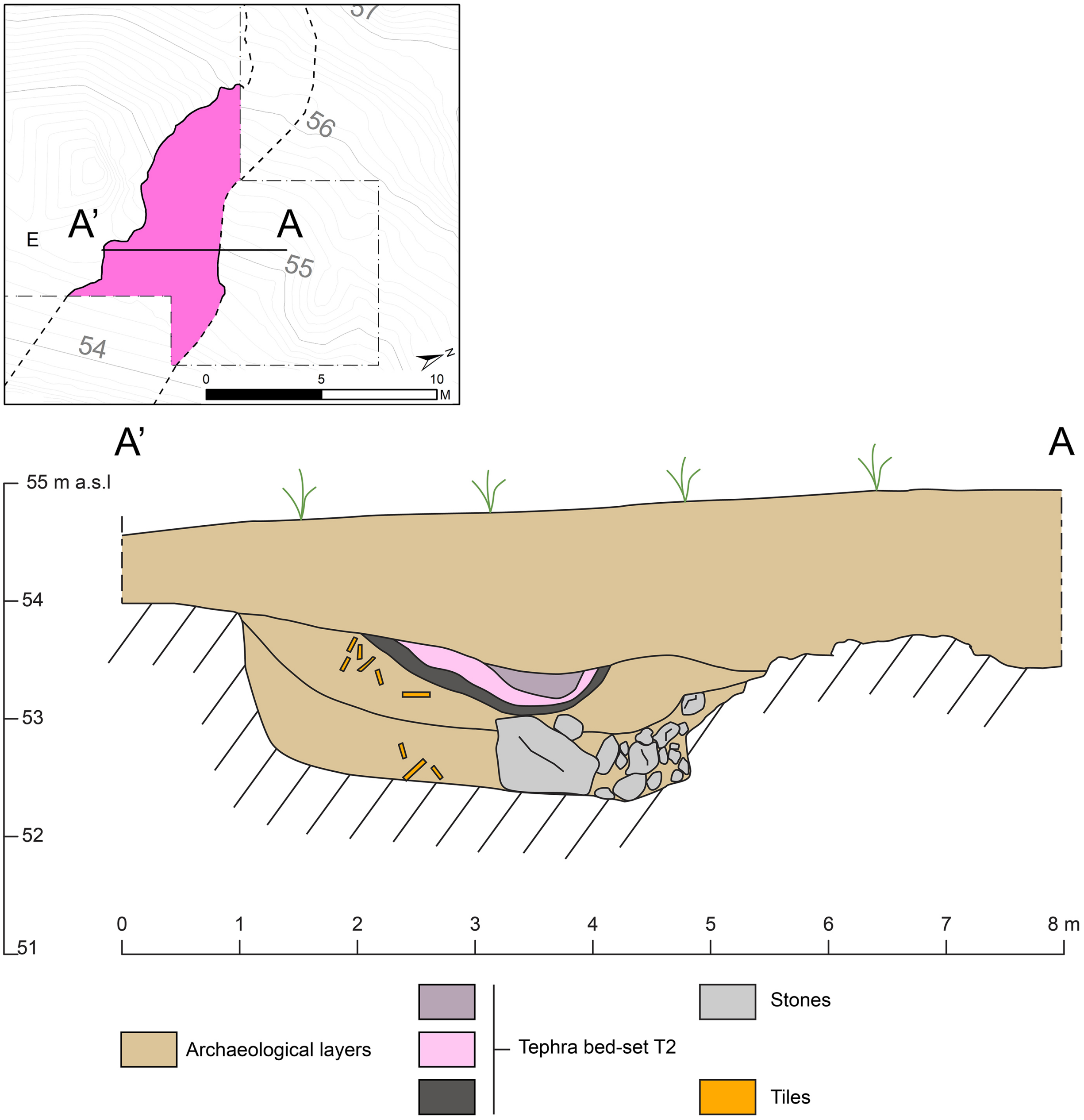

Figure S4.
Stratigraphy of the channel in the East area of the archaeological excavation.

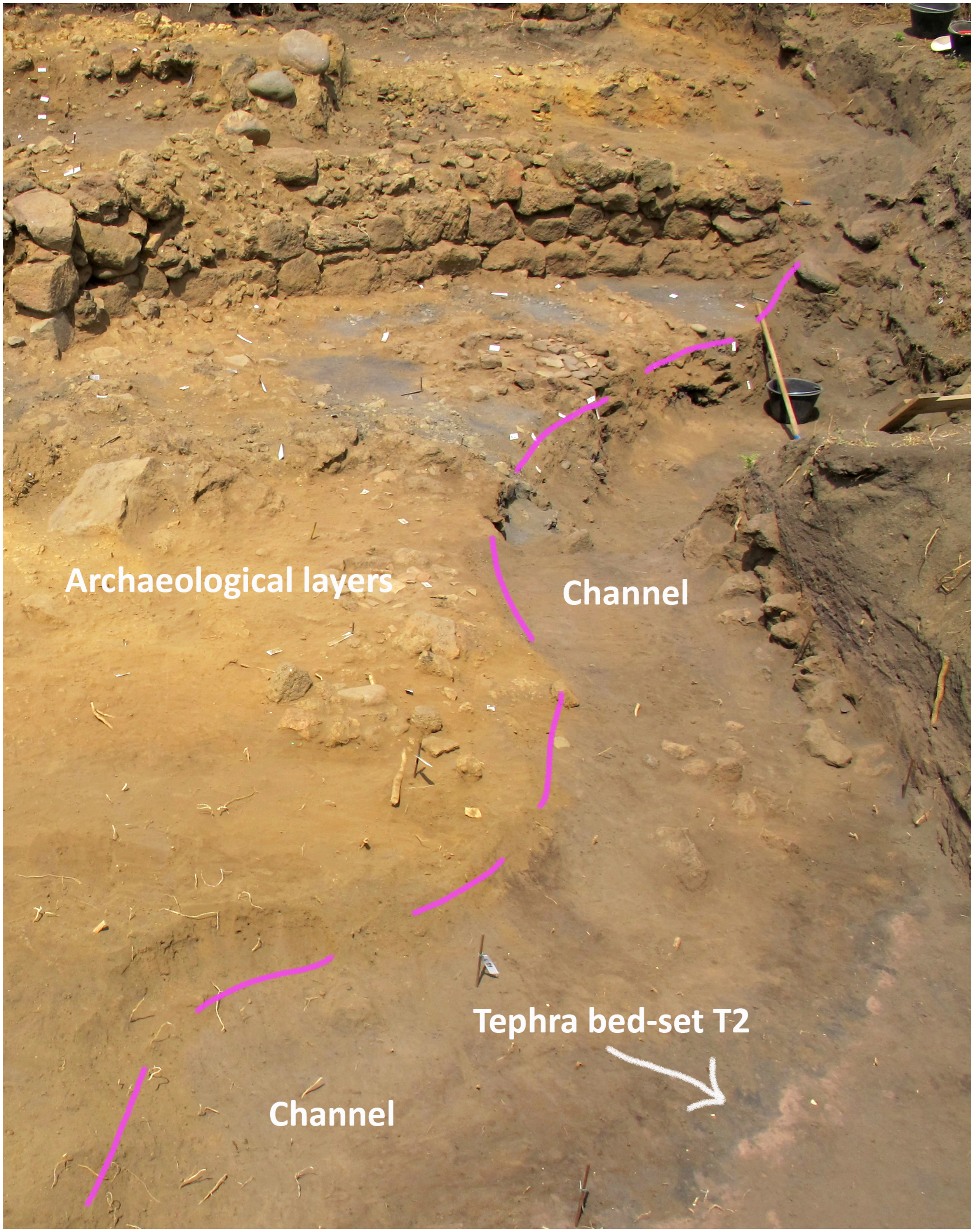

Figure S5.
Photo of the tephra T2 in the East area of the archaeological excavation.

Figure S6.

In the previous page, a 3D model of the medieval channel in the East area of the archaeological excavation.

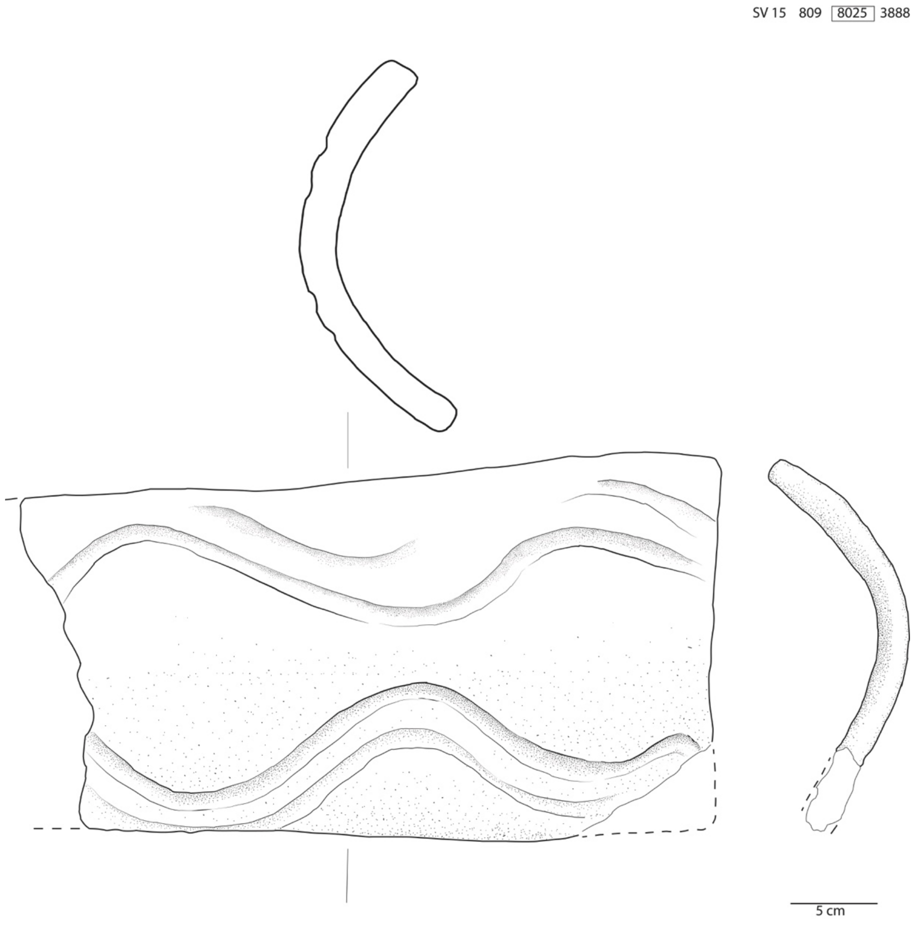

Figure S7.

Decorated tile with finger-impressed wavy lines from the medieval church (inv. 3888, drawing P. Vertuani). Chrono-typological comparisons are known in Sicily (Merì -ME) and Calabria (Santa Maria del Mare-Stalettì). In Sicily a 13th cent. date was suggested, although most associated objects would point to an earlier date in the 11th or 12th cent.; in Calabria the occupation phase is dated to the first half of the 11th cent.[1,2].

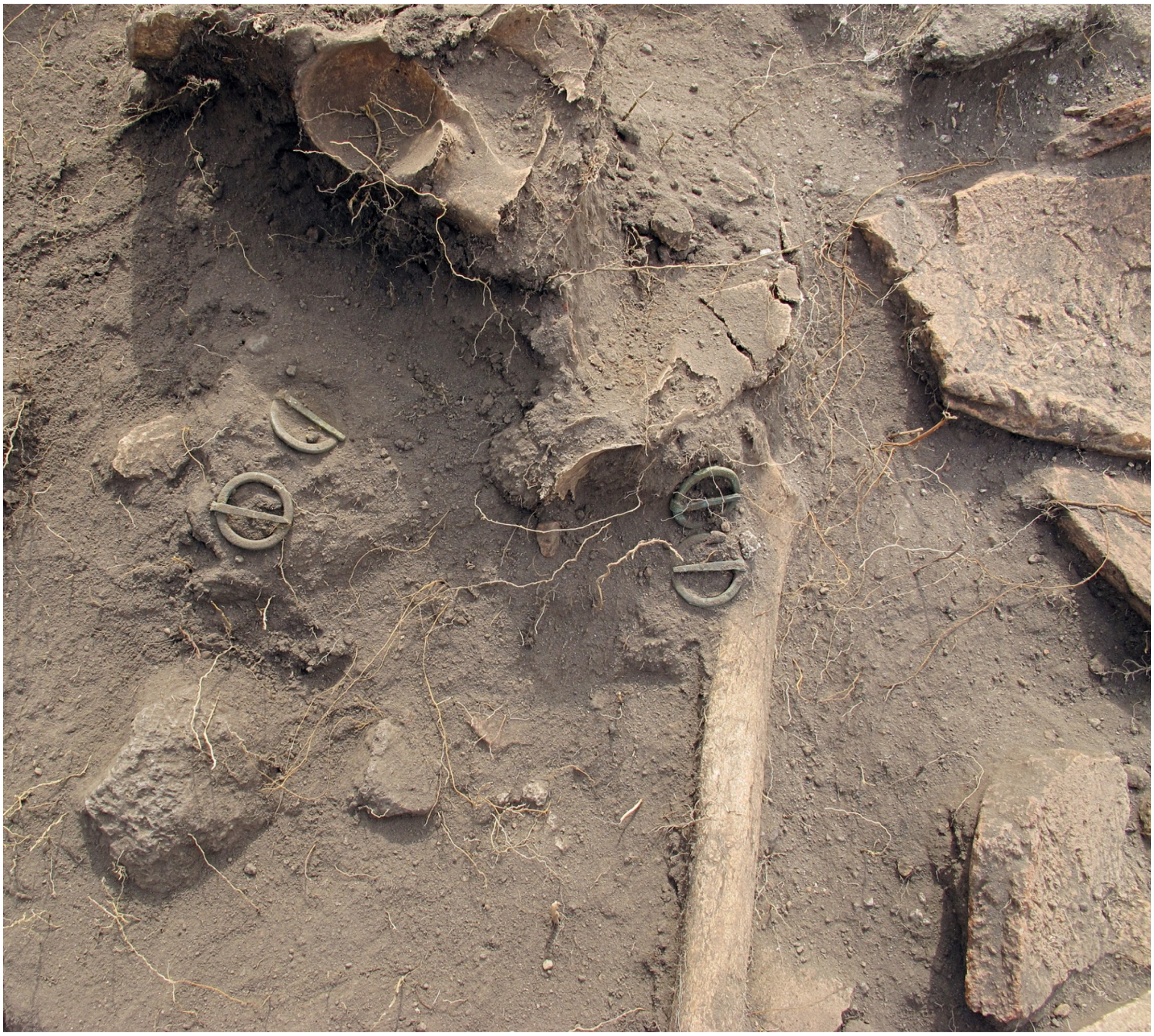

Figure S8.

Buckles discovered during the excavation of the grave 5, North area of the archaeological excavation.

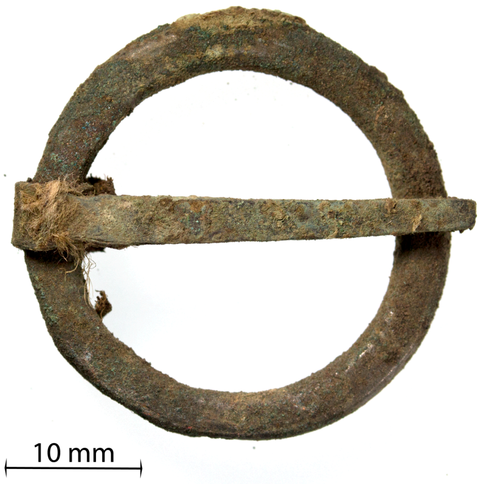

Figure S9.

Buckle from grave 5 (inv 4639): circular ring of round section with freely moving pin of rectangular section, 31-32 mm diameter, copper alloy. Close parallels have been found at Brucato in northwestern Sicily, where they were found amid domestic materials associated with an occupation that lasted from the late 13th to the mid-14th cent.[3]. Numerous parallels are also known from England, where they are dated between 1270 and 1450, especially the interval from 1350 to 1400[4].

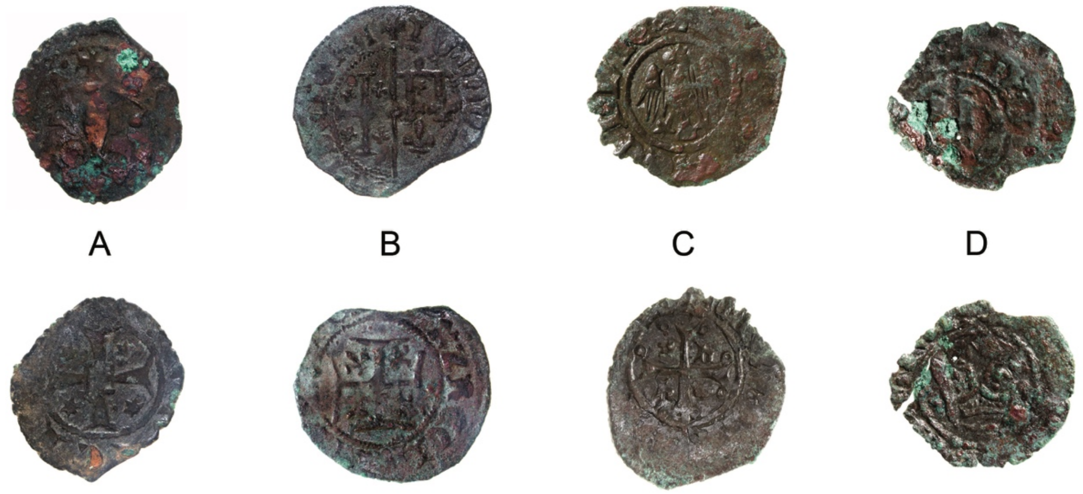

Figure S10.

Coins from the church area: (A) denaro of Charles I of Anjou (inv 3870-ME29392), who was king of Sicily from 1266 to 1285; (B) denaro from the joint reign of Joanna I and Louis of Naples (1352–1362) (inv. 3868-ME29390); (C) denaro of Palermo[5], produced during the minority of Maria of Sicily (c. 1377–c. 1380) (inv. 3809-ME29382); (D) denaro from the joint reign of Maria and Martin I of Sicily (1395–1402) (inv. 3802-ME29375).

TABLE S1. AMS RESULTS OF RADIOCARBON AGE DETERMINATIONS AND CALENDAR AGE CONVERSION (68.2% AND 95.4% PROBABILITIES). SAMPLING LOCATIONS AND STRATIGRAPHIC HEIGHTS ARE ALSO INDICATED.

Sample	Number (Figure 4)	Type of sample	Location	Stratigraphic height and SU	^{14}C age BP (± 1σ)	Calendar age AD 2σ (95.4%)	Calendar age AD 1σ (68.2%)
St16-71	19	Charcoal	Trench 4	Below black/gray ash	330±30	1477 – 1642	1540 – 1601 (37.4%)<br>1495 – 1530 (19%)<br>1616 – 1634 (11.8%)
1778	18	Charcoal	North area	Tephra, SU858	340±30	1470 – 1640	1557 – 1602 (30.7%)<br>1490 – 1525 (23.2%)<br>1610 – 1632 (14.3%)
1756	17	Charcoal	East area	Below tephra, SU2065	340±30	1450 – 1640	1470 – 1525<br>1555 – 1630
264*	16	Charcoal	East area	Below tephra SU209	420±40	1430 – 1520	1440 – 1470 (68.2%)
266	15	Charcoal	East area	Below tephra, SU207	463±32	1410 – 1470	1425 – 1449
St16-34	14	Charcoal	Trench 2	Below upper tsunami	420±30	1426 – 1515 (87.9%)<br>1598 – 1618 (7.5%)	1438 – 1479
1788	13	Charcoal	North area	Between tiles and tephra, SU868	530±30	1390 – 1440 (75.9%)<br>1320 – 1350 (19.5%)	1398 – 1432
265	11	Charcoal	East area	Channel below tephra, SU2022	514±33	1325 – 1445	1405 – 1435
1757	10	Human bones	North area	Grave 4, apse	550±30	1386 – 1434 (54.9%)<br>1311 – 1359 (40.5%)	1394 – 1420 (43.9%)<br>1326 – 1344 (24.3%)
1769	9	Human bones	North area	Grave 5, northern room	610±30	1295 – 1404	1302 – 1328 (27.5%)<br>1340 – 1367 (27.0%)<br>1382 – 1396 (13.7%)
1759	8	Charcoal	North area	Hole in apse's pavement, SU812	590±30	1285 – 1400	1295 – 1320<br>1350 – 1390
St16-82	5	Charcoal	Trench 1	Below lower tsunami	730±30	1224 – 1298	1262 – 1286

*M. Coltelli, pers. comm.

Table S1.

Results of radiocarbon age determinations and calendar age conversion (68% and 95% probabilities) AMS analyses, sampling locations, and sample stratigraphic heights and SU (stratigraphic unit).

TABLE S2. THERMOLUMINESCENCE RESULTS OF TILES SAMPLED IN THE ARCHAEOLOGICAL SITE.

Sample	Stratigraphic unit (SU)	Age (years AD)	Error (± years)
D2643a	C1785	1110	55
D2643b	C1785	1120	55
D2643c	C1785	1160	50
**Average**		**1130**	**50**
D2644a	C1786	1320	45
D2644b	C1786	1300	45
D2644c	C1786	-	-
**Average**		**1310**	**40**

Table S2.

Results of thermoluminescence measurements conducted at the laboratories of Milano Bicocca University (Italy). Datings are the average of 2/3 tiles sampled in situ with sediment.

Supplementary material references

1. Bonanno, C. *et al.*, "Merì: Località S. Giuseppe." In *Alle radici della cultura mediterranea ed europea: I Normanni nello Stretto e nelle isole Eolie*, G.M. Bacci and M.A. Mastelloni, Eds. Lipari: Museo Archeologico Regional Eoliano "Luigi Bernabò Brea", 86–92 (2002).
2. Donato, E. & Raimondo, C. Nota preliminare sull'utilizzo e la produzione di mattoni nella Calabria postclassica. I mattoni dallo scavo del *castrum* di S. Maria del Mare à Stalettì (CZ). *Mélanges de l'École française de Rome – Moyen Âge* 113, **1**, 173–20 (2001).
3. Piponnier, F. Les objets fabriqués autres que monnaies et céramique in Brucato: Histoire et Archéologie d'un Habitat Médiéval en Sicile, J.-M. Pesez, Ed. École Française de Rome., 497–614 (1984).
4. Egan, G. & Pritchard, F. Dress Accessories, c. 1150–c. 1450. *Medieval Finds from Excavations in London 3*. Woodbridge Boydell Press (2002).
5. Bonanno, M. Denaro inedito della città di Palermo di epoca chiaramontana, Schede Medievali 6, 92–98 (1984).
